# An Enhanced Lemaitre Model and Fracture Map for Cr5 Alloy Steel during High-Temperature Forming Process

**DOI:** 10.3390/ma15113935

**Published:** 2022-05-31

**Authors:** Xuewen Chen, Lele Guo, Bo Zhang, Rongren Bai

**Affiliations:** School of Materials Science and Engineering, Henan University of Science and Technology, 263 Kaiyuan Avenue, Luoyang 471023, China; 18438603059@163.com (L.G.); zhangbo32103@126.com (B.Z.); bairongren317@163.com (R.B.)

**Keywords:** Cr5 alloy steel, Lemaitre damage model, Zener–Hollomon coefficient, elastic modulus, fracture map

## Abstract

To effectively control and predict crack defects in the high-temperature forming process of Cr5 alloy steel, based on the traditional Lemaitre damage model, a new high-temperature damage model of Cr5 alloy steel was proposed which considered the change of material elastic modulus with temperature, the influence of material hydrostatic pressure as well as temperature and strain rate on material damage. Because Cr5 alloy steels are usually forged at high temperatures, tensile testing is an important method to study the damage behaviour of materials. Through the high-temperature tensile test and elastic modulus measurement test of the Cr5 alloy steel, the stress–strain curves and the relationship curves of the elastic modulus value with the temperature of Cr5 alloy steel under different temperatures and strain rates were obtained. A new high-temperature damage model of Cr5 alloy steel was built by introducing the Zener–Hollomon coefficient considering the influence of temperature and strain rate. The established high-temperature damage model was embedded in Forge^®^ finite element software through the program’s secondary development method to numerically simulate the experimental process of Cr5 alloy steel. Comparing the difference between the displacement–load curves of the numerical simulation and the actual test of the tensile process of the experimental samples, the correlation coefficient R^2^ is 0.987 and the difference between the experimental value and the simulated value of the tensile sample elongation at break is 1.28%. The accuracy of the high-temperature damage model of Cr5 alloy steel established in this paper was verified. Finally, the high-temperature damage map of Cr5 alloy steel was constructed to analyse the variation law of various damage parameters with the temperature and strain rate of the high-temperature damage model of Cr5 alloy steel.

## 1. Introduction

As the key components of the rolling mill of the steel rolling production line, with the continuous development of the steel rolling industry, the requirements for the hardness and wear resistance of the rolls in industrial production are getting higher and higher. Cr5 alloy steel has been widely used in the production of modern rolls as a substitute for 60CrMnMo, 50CrNiMo, 70Cr3NiMo and Cr4 [1] due to its good hardness, wear resistance and resistance. Fatigue performance has always been one of the key research topics for roll steels in the iron and steel production industry. Many scholars from all over the world have conducted a lot of research into the mechanical properties and fatigue resistance of Cr5 alloy steel and have achieved certain results. Junkai Fan et al. [2] determined the forming properties of Cr5 alloy steel by studying the mechanical properties of Cr5 alloy steel. Qing Gu et al. [3] determined the mechanical properties of Cr5 alloy steel by studying the fatigue behaviour of Cr5 alloy steel. M. Surendran [4] and others determined the fatigue characteristics of Cr5 alloy steel by studying the hoop stress of Cr5 alloy steel. Many scholars conducted in-depth research on the mechanical properties of Cr5 alloy steel, which are beneficial to actual industrial production to a certain extent. However, there have been few studies on the damage performance of this material. Therefore, the current hot forming production plan for Cr5 rolls is often made by trial and error based on actual production experience, and there is no effective method to control crack defects.

Damage mechanics is a discipline that studies the process of material damage evolving with deformation until it is destroyed. It is believed that there are micro-defects distributed inside the material, such as dislocations, micro-cracks, micro-voids, etc. These micro-structures of different scales are typical damage performance [5]. Y.C. Huang et al. studied the damage mechanism of 42CrMo steel and found that the growth and accumulation of pores were the main reasons for alloy fracture [6]. Y.C. Lin et al. studied the fracture mechanism of nickel-based superalloys. They found that the higher the nucleus density formed by micropores is, the worse the plastic deformation ability of the material is, and that the combination of micropores was one of the reasons for the final ductile fracture [7]. Kaimeng Wang et al. conducted tensile experiments on nickel-based superalloys and found that pores were mainly formed around grain boundaries and carbides in grains, and the fracture morphologies are characterized by transgranular fracture and intergranular fracture [8]. Many scholars have conducted in-depth research on the formation mechanism of material cracks which is beneficial to reduce or eliminate the initiation of material damage during plastic deformation. However, no damage model was used to describe the damage behaviour of the materials.

In recent decades, many scholars have performed systematic research on the mechanism of crack generation and the establishment of related models in the material forming process. Freudenthal [9] believed that when the material strain energy reached a critical value, the material would fracture, and proposed the Freudenthal damage model. Cockcroft and Latham [10] believed that the maximum tensile stress in the material deformation process was the cause of material fracture, and proposed the Cockcroft and Latham (CL) damage model. Oyane [11] believed that the pores were produced by the large deformation or the influence of the second phase particles, and proposed the Oyane damage model which considers stress triaxiality. Weitao Jia [12] used the Freudenthal damage model to study the deformation and fracture behaviour of as-cast AZ31B Mg alloy. Qiang Li [13] used the Cockroft and Latham fracture criterion to study the surface cracking of Fe-Cu-C steel. Feuerhack et al. [14] used the Cockcroft and Latham damage criterion to study crack location and shape during extrusion. Aditya Rio Prabowo et al. [15] analysed the reliability of the Cockcroft and Latham damage criterion. Wu Ying et al. [16] used the Oyane fracture criterion to compare and analyse the dent defect failure of the X80 pipeline; Yu Zhang et al. [17] used the Oyane fracture criterion to study the damage and cracking characteristics of AISI 410 stainless steel at high-temperature. Zbigniew Pater [18] used the Oyane damage criterion to systematically study the critical damage value of 100Cr6 steel. Many scholars used the above damage models to effectively predict the damage behaviour during the material forming process which played a guiding role in controlling the damage defects in production. However, these damage models use the correlation function of strain and stress to calculate the damage parameters, and the prediction accuracy is slightly lower [19].

The Lemaitre damage model in damage mechanics was proposed based on the thermodynamic dissipation potential of the material. This phenomenological method has been used to describe the damage of the material, which is closer to the real physical situation, and can more accurately describe the damage evolution behaviour of the material. Manizheh Aghaei et al. [20] studied the tensile process of DP600 steel based on the Lemaitre damage model, and described the micromechanical behaviour of DP600 steel under tensile load. Based on the Lemaitre damage model, Sheng Cai et al. [21] studied the blanking process of T6, and the damage of the material during the blanking process was simulated. Yogeshwar Jasra et al. [22] studied the cracking process of AISI 304 stainless steel based on the Lemaitre damage model, and obtained the failure characteristics of AISI 304 stainless steel. Ashwani Verma et al. [23] simulated the tensile process of the dual-phase steel based on the Lemaitre damage model, and obtained the damage parameters of the dual-phase steel in the tensile state. Based on the Lemaitre damage model, the fatigue life of the alloy was studied and predicted by L.M. Araújo et al. [24]. Kazem Malekipour et al. [25] observed and simulated the damage evolution process of AISI 316L parts based on the Lemaitre damage model. The axial stretching process was simulated, and the damage evolution law of the thin-walled steel pipe was obtained by Sheng He et al. [26]. Many scholars have used the traditional Lemaitre damage model to accurately predict the damage behaviour of different forming processes. Tandon Puneet et al. [27] simulated the establishment of progressive sheet metal forming process based on the Lemaitre damage model and investigated the effectiveness of the Lemaitre damage model in the simulation of the ISF process. However, this model can only be used for the calculation and prediction of damage defects in the cold forming process of metal materials. Because this model does not affect the crack initiation and propagation process of temperature and strain rate during the high-temperature forming process, it cannot be used for the calculation and prediction of damage defects in the high-temperature forming process; On the other hand, this damage model lacks consideration of the effect of temperature-induced elastic modulus changes on material damage [28].

The traditional Lemaitre damage model does not consider the effect of temperature and strain rate on damage, and it is difficult to predict damage behaviour at high temperature. In this paper, considering the influence factors of temperature and strain rate, the high-temperature Lemaitre damage model of Cr5 alloy steel is improved and established, and its high temperature damage map is drawn to accurately predict the damage initiation of Cr5 alloy steel at high temperature.

In order to study the damage law of Cr5 alloy steel at high temperature, the hot tensile test and high temperature elastic modulus measurement test of Cr5 alloy steel were carried out. Based on the test data, a high-temperature Lemaitre damage model of Cr5 alloy steel considering the temperature and strain rate was established to predict its high-temperature damage behaviour. The high-temperature damage model of Cr5 steel was embedded in the finite element software FORGE^®^ to simulate the tensile test of the Cr5 alloy steel. The accuracy of the established high-temperature damage model was verified by comparing the displacement–loads and fracture lengths of the simulation and the test. In order to avoid the crack defect of the Cr5 roll during hot forming, the corresponding Lemaitre high-temperature damage model damage map was proposed.

## 2. Materials and Test Methods

### 2.1. Materials and Test Procedures

**Figure 1:**
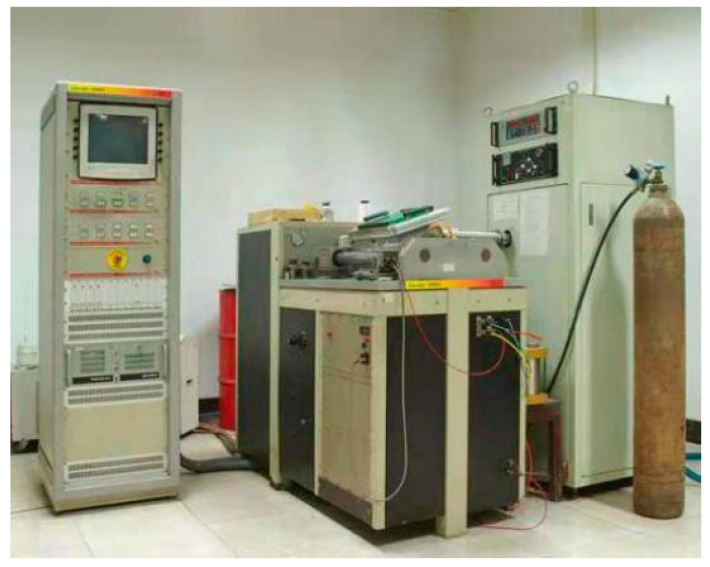
Gleeble-1500D thermal simulation testing machine.

**Figure 2:**
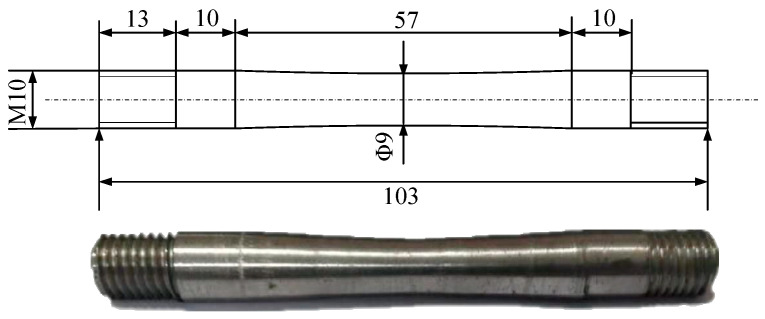
Schematic drawing of specimens for tensile tests (unit: mm).

The material used in the test is Cr5 alloy steel, which is used in conventional industrial production and its chemical compositions are shown in Table 1. The equipment used in the test is a Gleeble-1500D thermal simulation testing machine, as shown in Figure 1. The machine has the function of the real-time calculation of strain, meaning that the strain ε equals ln(L/L0) in real time. In a certain time interval delta t, the strain changed delta ε, then under the linear control of the strain, a strain rate equal to (delta ε)/(delta t) and equal to a constant can be achieved. The central part of the tensile specimen is processed by means of arc transition to ensure that the tensile specimen can be smoothly broken from the central part [29]. The samples have a taper that gradually decreases from the end to the centre, and both ends are stretched with M10 standard thread. The specific shape and size of the tensile specimen are shown in Figure 2, which was prepared in accordance with ASTM E8/E8M-2016a.

Because 800 °C–1150 °C is the common forging temperature range of the Cr5 roll, and the strain rate of the material ranges between 0.01 s^−1^ and 5 s^−1^ in actual production, the temperatures used in the test are 800 °C, 850 °C, 900 °C, 950 °C, 1000 °C, 1050 °C, 1100 °C, and 1150 °C, and the real strain rates are set as 0.01 s^−1^, 0.1 s^−1^, 1 s^−1^, 5 s^−1^ by Gleeble-1500D thermal simulation testing machine, and a tensile test is performed at each temperature and strain rate, and the total number of samples consumed are 32. Using thermocouple wire to measure the temperature of the tensile sample, the tensile specimen was first heated to the specified temperature at a speed of 10 °C/s, and then kept warm for 3 min to eliminate the temperature gradient. Finally, it remained tensile at a predetermined strain rate until fracture.

### 2.2. Analysis of Test Results

**Figure 3:**
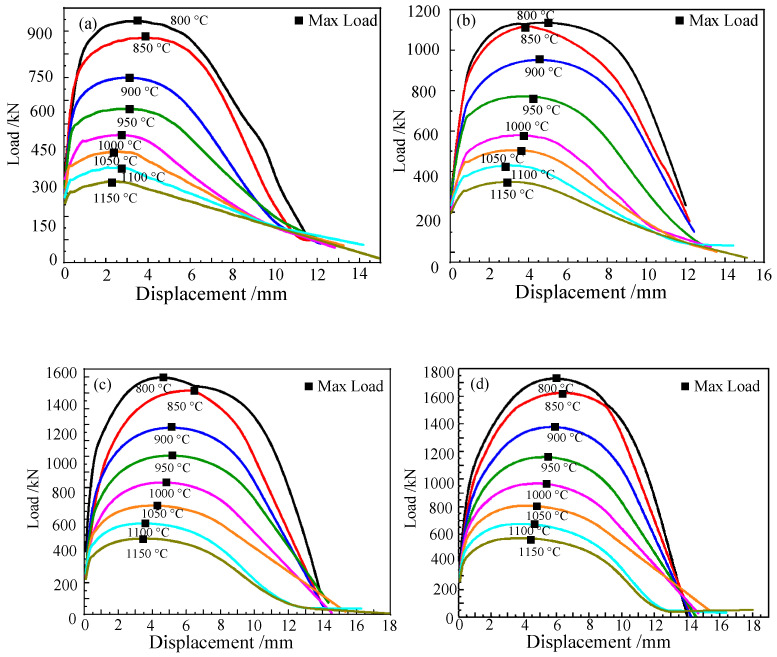
Cr5 steel displacement–load curves with different temperatures and different strain rates: (**a**) $\dot{\varepsilon }$ = 0.01 s^−1^; (**b**) $\dot{\varepsilon }$ = 0.1 s^−1^; (**c**) $\dot{\varepsilon }$ = 1 s^−1^; (**d**) $\dot{\varepsilon }$ = 5 s^−1^.

Figure 3 shows the displacement–load curves of the Cr5 alloy steel obtained by tensile tests. Table 2 shows the fracture displacement of the Cr5 alloy steel at different temperatures and strain rates. It can be seen from the figure that at the same strain rate, with the continuous increase in temperature, the fracture displacement of Cr5 alloy steel gradually increases. When the rate is 1 s^−1^, the fracture displacement of Cr5 alloy steel is 13.89 mm at 800 °C, and the fracture displacement of Cr5 alloy steel is 17.93 mm at 1150 °C. For example, when the temperature is 1000 °C, the fracture displacement of Cr5 alloy steel is 12.83 mm when the strain rate is 0.01 s^−1^, while the fracture displacement of Cr5 alloy steel is 14.69 mm when the strain rate is 5 s^−1^. At the same strain rate, with the continuous increase in temperature, the maximum load of Cr5 alloy steel during stretching gradually decreases. For example, when the strain rate is 5 s^−1^, the maximum load of Cr5 alloy steel when stretched at 800 °C is 1707.44 N and the maximum load of Cr5 alloy steel in tension is 571.53 N at 1150 °C. At the same temperature, with the gradual increase in strain rate, the maximum load of the Cr5 alloy steel during stretching gradually increases. Additionally, when the strain rate is 5 s^−1^, the load is 250.52 N, and the maximum load when the Cr5 alloy steel is stretched is 571.53 N. The experimental results are consistent with the literature [30]. It can be obtained that the maximum load of Cr5 alloy steel material can be reached when plastic deformation occurs and decreases with the increase in temperature, and its maximum load increases with the increase in strain rate. Additionally, the effect of temperature on the maximum load of Cr5 alloy steel when plastic deformation occurs is greater than the effect of the strain rate. This is because, with the continuous increase in temperature, the Cr5 alloy steel material will recover and recrystallize at high-temperature, and the maximum load required for plastic deformation will gradually decrease due to the softening of the material. However, with the increase in strain rate, the maximum load required for the plastic deformation of Cr5 alloy steel will gradually increase with the increase in the strain rate due to the influence of the deformation degree per unit time [31].

Therefore, in the actual production of Cr5 alloy steel products and the formulation of the hot forming process plan, in order to effectively control crack defects, the highest temperature and a lowest strain rate possible should be selected.

## 3. Establishment of High-Temperature Damage Model of Cr5 Alloy Steel

### 3.1. Traditional Lemaitre Damage Model

Damage is a very important factor affecting the quality of metal products. However, studying the damage of a specific material is a very complicated process, which is closely related to the stress state of the material during plastic processing [32]. Therefore, it is of great significance to determine a more reliable material damage model to effectively predict and control the product crack defects.

The Lemaitre damage model is a damage model that clearly expresses the damage of materials and considers more parameters and variables, and can be directly applied to guide the production and manufacture of products in industrial production. The traditional Lemaitre damage model considers the material to be damaged or destroyed when the absolute damage value *D* suffered by the material during processing is greater than or equal to the critical damage value *D_c_* of the material itself, and its mathematical formula is expressed as follows [33]:

(1) $$dD=\frac{f\left(\xi \right){\bar{\sigma }}^{2}}{2ES{\left(1-D\right)}^{2}}d\bar{\varepsilon }$$

(2) $$f\left(\xi \right)=\left[\frac{2}{3}(1+v)+3(1-2v){\left(\frac{\sigma_{m}}{\bar{\sigma }}\right)}^{2}\right]$$

where *dD* represents the absolute damage value increment; f(ξ) represents the stress triaxiality function; ξ represents the stress triaxiality; *E* represents the elastic modulus of the material; *S* represents the anti-damage factor; ε represents the equivalent plastic strain; $d\bar{\varepsilon }$ represents the equivalent plastic strain increment; *v* is the Poisson’s ratio of the material; *σ_m_* represents the mean stress; *σ* represents the equivalent stress experienced by the material.

However, in actual industrial production, the stress state experienced by materials is often not in a single direction, but in multiple axial stress states. Therefore, the expression of *D_c_* in the Lemaitre damage model under multiaxial stress states is as shown in Formula (3):

(3) $$D_{c}=D_{1c}\frac{s_{U}{}^{2}{\left(1-D\right)}^{2}}{f\left(x\right){\bar{s}}^{2}}$$

where *σ_u_* and *D*_1*c*_ represent the stress limit value and the damage critical value of the material in the tensile test, respectively.

The damage variable *D* represents the damage degree of the material in the unit. When the value of the damage variable *D* is 0, there is no damage inside the material. When the damage variable of the material reaches the critical damage value *D_c_*, this means that the material is damaged. In the Lemaitre damage model, the relative damage value *D_rel_*, that is, the ratio of the absolute damage value *D* to the critical damage value *D_c_*, is used to represent the degree of damage inside the material [34], and its specific expression is as follows:

(4) $$D_{rel}=D/D_{c}(0\leq D_{rel}\leq 1)$$

All four damage parameters *D*_1*c*_, *σ_u_*, *S* and *ε_th_* in the Lemaitre damage model can be obtained through the tensile test of the material, and the damage parameters at different temperatures and different strain rates that can be obtained through the actual tensile test data according to Formulas (5)–(8) are calculated:

(5) $$\varepsilon_{th}=\varepsilon_{m}$$

(6) $$\sigma_{U}=R_{m}$$

(7) $$D_{1c}=1-R_{B}/R_{m}$$

(8) $$S=\frac{{\sigma_{U}}^{2}(\varepsilon_{B}-\varepsilon_{m})}{2ED_{1c}}$$

where *ε_m_* is the tensile strain of the material; *ε_B_* is the fracture strain of the material; *R_m_* is the tensile strength of the material; *R_B_* is the breaking strength of the material.

### 3.2. Modification of Lemaitre Damage Model

Without considering the effect of temperature, the traditional Lemaitre damage model cannot be applied to the high-temperature forming process of the material, and the stress triaxiality term in the damage model is a square term, which is not able to distinguish between the influence of positive and negative hydrostatic stress on the material damage. Therefore, improvements and corrections are required. The Zener–Hollomon equation is a function related to temperature and strain rate, which can describe the rheological behaviour of materials at different temperatures and strain rates. Its specific expression is shown in Equation (9) [35]:

(9) $$Z=\dot{\varepsilon }\exp\left[Q/RT\right]$$

where $\dot{\varepsilon }$ represents the strain rate of the material; Q represents the deformation activation energy of the material; *R* is the gas constant; *T* is the deformation temperature of the material.

In previous experimental studies [36,37], it was found that compressive stress often plays a role in the healing of micro-defects and micro-cracks in the material in the process of plastic deformation, and there is a certain difference with the role of tensile stress in the process of material damage. The traditional Lemaitre damage model does not fully consider the different effects of tensile and compressive stress on the damage of the material. Thus, the damage model often cannot accurately describe the damage of the material. Therefore, the influence of the internal compressive stress of the material on the damage of the material (which will promote crack healing) can be removed through coupling, and the Lemaitre damage model of the Cr5 alloy steel with only tensile stress is obtained. The traditional Lemaitre damage model is modified to obtain the modified Lemaitre damage model [38].

The absolute damage value increment equation of the modified Lemaitre damage model is:

(10) $$dD=\{\begin{array}{cc} \begin{array}{c} 0\\ \frac{f\left(\xi \right){\bar{\sigma }}^{2}}{2ES{\left(1-D\right)}^{2}}d\bar{\varepsilon } \end{array} & \begin{array}{c} \begin{array}{l} \sigma_{m}\leq 0\;or\;\bar{\varepsilon }\leq \varepsilon_{th}\\ \sigma_{m}>0\;or\;\bar{\varepsilon }>\varepsilon_{th} \end{array} \end{array} \end{array}$$

(11) $$f\left(\xi \right)=\left[\frac{2}{3}(1+v)+3(1-2v){\left(\frac{\sigma_{m}}{\bar{\sigma }}\right)}^{2}\right]$$

In order to obtain the Lemaitre damage model of Cr5 alloy steel suitable for different temperatures and different strain rates, the Zener–Hollomon coefficient was coupled with the traditional Lemaitre damage model, and the damage parameter values of the Cr5 alloy steel at different temperatures and different strain rates were obtained. As such, a Lemaitre damage model of the Cr5 alloy steel which is suitable for different temperatures and different strain rates can be built, the Lemaitre damage model can be coupled using the Zener–Hollomon coefficient, and it can be used to manufacture Cr5 alloy steel products at different temperatures and strain rates in industrial production [39].

In summary, the coupled form of the damage parameters in the improved Lemaitre damage model based on the Zener–Hollomon coefficient are:

(12) $$Z=\dot{\varepsilon }\exp\left[Q/RT\right]$$

(13) $$\varepsilon_{th}=f_{\varepsilon_{th}}\left(\ln Z\right)$$

(14) $$S=f_{s}\left(\ln Z\right)$$

(15) $$D_{1c}=f_{D_{1c}}\left(\ln Z\right)$$

(16) $$\sigma_{U}=f_{\sigma_{U}}\left(\ln Z\right)$$

As a model that can predict the damage of materials, the Lemaitre damage model is closely related to various parameters of the material. As a discipline that describes the damage of materials, damage mechanics are related to the properties of all aspects of materials. As a physical quantity describing the elastic properties of materials, the elastic modulus *E* is a very fundamental parameter variable in the Lemaitre damage model. The value of the elastic modulus *E* used in the calculation of the previous Lemaitre damage model is often a constant, and the elastic modulus *E* value of the material at different temperatures is not measured. The Gleeble-1500D thermal simulation testing machine used in this paper used C-Gauge sensor to accurately measure the elastic modulus *E* values of Cr5 alloy steel at different temperatures, and these values can be substituted into the Lemaitre damage model so as to obtain a more accurate Lemaitre damage model of the Cr5 alloy steel at different temperatures and different strains.

### 3.3. Measurement of Elastic Modulus E Value

As one of the essential performance parameters of engineering materials, the modulus of elasticity’s value will fluctuate with different material composition and structure. Especially as the temperature of the material increases, the distance between atoms inside the material will gradually increase and then the bond between atoms will be weakened, resulting in a decreased *E* value, higher temperature, and greater changes in the value of the elastic modulus. The elastic modulus *E* values of the same material at room temperature and at high temperature are often quite different. Therefore, in the present paper, the study of the damage of a certain material at high-temperature was performed and the modulus of elasticity is a crucial factor.

The elastic modulus is a physical quantity that describes the elastic properties of materials. The value of the elastic modulus *E* of the material is quite different under different conditions. Especially under the influence of material temperature, it often changes several times or even dozens of times. It has a great influence on establishing an accurate high-temperature damage model. For Cr5 alloy steel, the elastic modulus value at room temperature is 206 GPa. However, with the temperature of the material rising to a higher temperature, its elastic modulus value sharply decreases. Therefore, this has great influence on the establishment of an accurate high-temperature Lemaitre damage model for Cr5 alloy steel [40]. Previous studies on the Lemaitre damage model have rarely involved attention to the change of material elastic modulus, the research results obtained are often quite different from the actual results and are of low accuracy in terms of material damage prediction. Therefore, in order to improve the accuracy of the high-temperature Lemaitre damage model of Cr5 alloy steel, it is necessary to measure the actual elastic modulus *E* value of Cr5 alloy steel at different temperatures.

**Figure 4:**
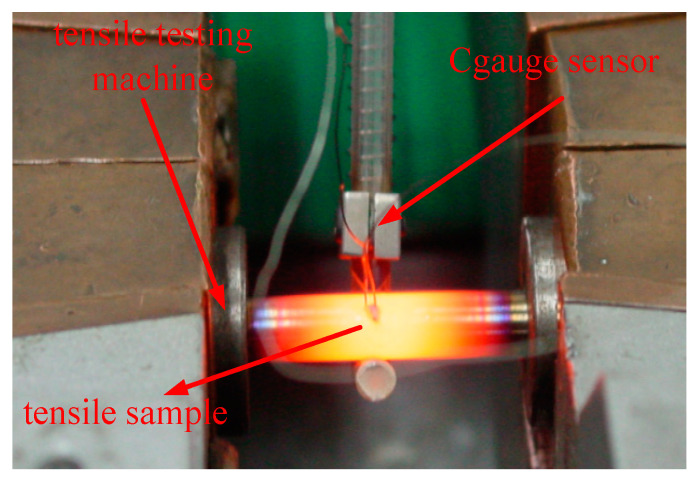
DSI C-Gauge sensor.

In this study, the elastic modulus *E* values of Cr5 alloy steel at different temperatures were measured. The measurement tests were performed on a Gleeble-1500D thermal simulation testing machine; the elastic modulus of Cr5 alloy steel was measured at different temperatures for one time each; the samples were heated at 10 °C /s and kept for 3 min to reach the specified temperature; the elastic modulus was then measured at the strain rate of 0.01 s^−1^; and the elastic modulus *E* values of Cr5 alloy steel at different temperatures were measured with the help of the C-Gauge sensor of the DSI company in the United States. The specific measurement results are shown in Table 3, whilst the fitting curve of the elastic modulus *E* value of Cr5 alloy steel and temperature is shown in Figure 4.

**Figure 5:**
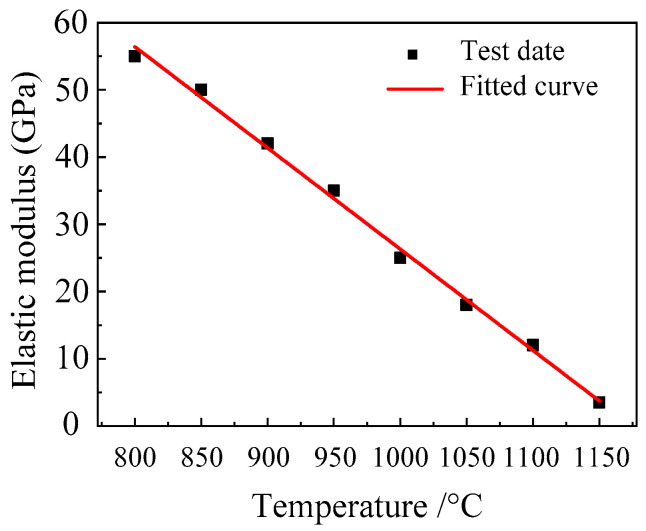
Fitting curve between the *E* value of the elastic modulus and temperature of the Cr5 alloy steel.

It can be seen from Figure 5 that the change curve of the elastic modulus *E* value of Cr5 alloy steel in the high-temperature stage with temperature is approximately a linear function. Therefore, it can be fitted using a linear function. The relationship curve of the elastic modulus of the Cr5 alloy steel at the high-temperature stage with temperature is obtained as follows:

(17) E=−0.15T+176.89

From the analysis of test results, it can be obtained that the elastic modulus *E* value of Cr5 alloy steel is 55 GPa under the condition of 800 °C and it is only 3.5 GPa at 1150 °C. These are quite different from the elastic modulus of 206 GPa of Cr5 alloy steel at room temperature. Additionally, the fitting function of temperature and elastic modulus has a high linear relationship, and the elastic model at other temperatures can be calculated according to Equation (17). This shows that after the increase in the temperature of Cr5 alloy steel, the atomic spacing inside the material increases, the thermal motion of the molecules intensifies, and then the ability to resist external elastic deformation is weakened.

**Figure 6:**
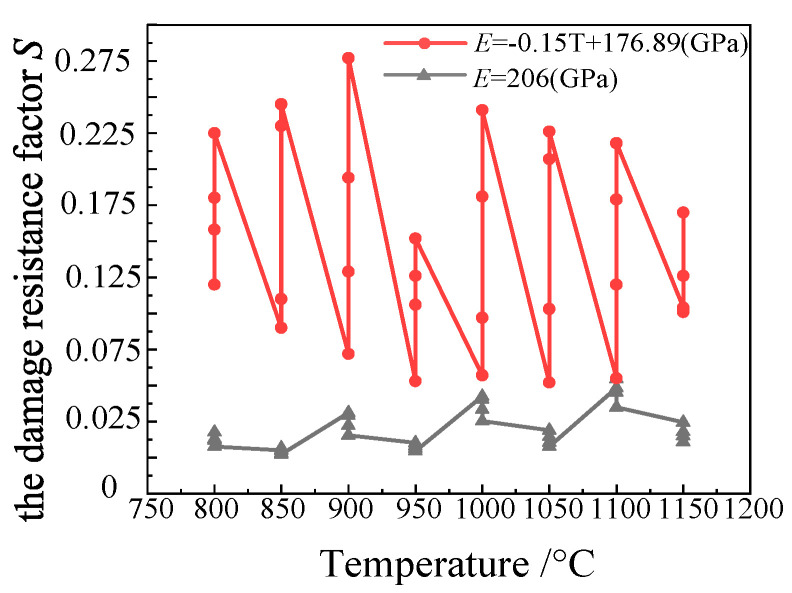
Comparison of the damage parameter *S* values of Cr5 alloy steel with different elastic moduli.

Figure 6 is a comparison diagram of the damage resistance factor *S* value of the Lemaitre damage model calculated by substituting the elastic modulus (206 GPa, red) of the Cr5 alloy steel at room temperature and the elastic modulus *E* value measured by the test (black). This figure shows that the damage resistance factor *S* value directly calculated by the constant elastic modulus of 206 GPa shows little difference at each temperature. Additionally, at the same temperature, the difference of the damage resistance factor *S* value under each strain rate is not obvious. However, the *S* value of the damage resistance factor calculated using the *E* value measured by the actual elastic modulus measurement test shows obvious differences at each temperature. Additionally, at the same temperature, the value of the damage resistance factor *S* at each strain rate is also quite different. This shows that the value of the elastic modulus *E* has great influence over obtaining an accurate material damage model. A more accurate Lemaitre damage model for the Cr5 alloy steel at different temperatures and strain rates can be obtained incorporating the *E* value measured by the actual elastic modulus measurement test.

### 3.4. Determination of Damage Model Parameters

The stress and strain data of the Cr5 alloy steel at different temperatures and strain rates can be obtained from the tensile tests. The Lemaitre damage parameters and Zener–Hollomon coefficient values of the Cr5 alloy steel at different temperatures and strain rates can be solved by Formulas (5)–(9). The calculation results are shown in Table 4.

### 3.5. Lemaitre High-Temperature Damage Model of Cr5 Alloy

**Figure 7:**
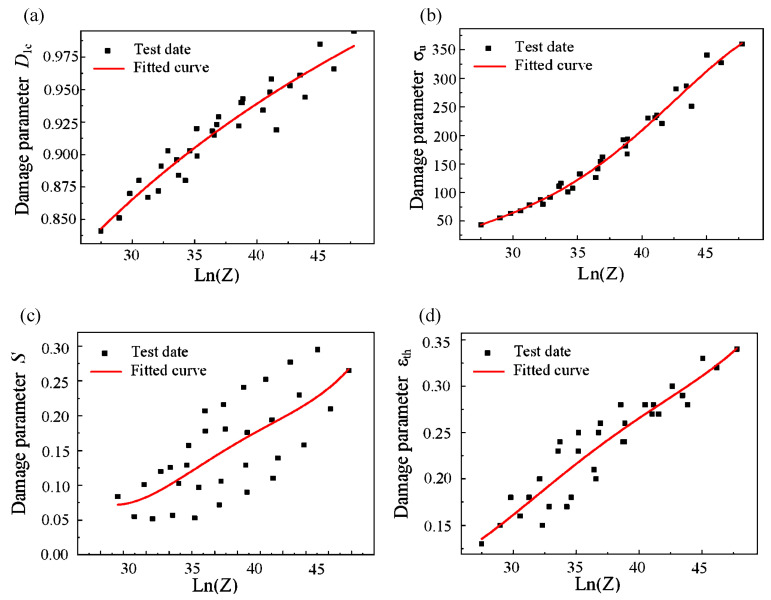
Fitting curve of coupling of Lemaitre damage model and Zener–Hollomon coefficient: (**a**) *D*_1*c*_-lnZ; (**b**) *σ_u_*-lnZ; (**c**) *S*-lnZ; (**d**) *ε_th_*-lnZ.

Coupling the Lemaitre damage model with the Zener–Hollomon coefficient, a Lemaitre high-temperature damage model of Cr5 alloy steel was founded. The damage parameters in Table 4 were polynomial fitted with ln(Z) based on the least square method. The fitting parameters of the obtained Lemaitre damage model coupled with the Zener–Hollomon coefficient are shown in Table 5. The fitted curve is shown in Figure 7.

It can be seen from Figure 7c that the fitting of anti-damage factor *S* is greatly dispersed, which may be caused by the difference in the influence degree of temperature and strain rate on *S*. Finally, the high-temperature damage model of the Cr5 alloy steel Lemaitre can be obtained:

(18) $$\left\{ \begin{array}{l} dD=\{\begin{array}{cc} \begin{array}{c} 0\\ \frac{f\left(\xi \right){\bar{\sigma }}^{2}}{2ES{\left(1-D\right)}^{2}}d\bar{\varepsilon } \end{array} & \begin{array}{c} \begin{array}{l} \sigma_{m}\leq 0\;or\;\bar{\varepsilon }\leq \varepsilon_{th}\\ \sigma_{m}>0\;or\;\bar{\varepsilon }>\varepsilon_{th} \end{array} \end{array} \end{array}\\ f\left(\xi \right)=\left[\frac{2}{3}(1+v)+3(1-2v){\left(\frac{\sigma_{m}}{\bar{\sigma }}\right)}^{2}\right]\\ {ƒ}_{\varepsilon th}(lnZ)=1.27-0.14lnZ+0.006{\left(lnZ\right)}^{2}-7.997E-5{\left(lnZ\right)}^{3}+5.81E-9{\left(lnZ\right)}^{4}\\ {ƒ}_{S}(lnZ)=5.48-0.61lnZ-0.025{\left(lnZ\right)}^{2}-4.43E-4{\left(lnZ\right)}^{3}+2.94E-6{\left(lnZ\right)}^{4}\\ {ƒ}_{D1c}=(1.59*0.13*{\left(lnZ\right)}^{0.66})/(1+0.13*{\left(lnZ\right)}^{0.66})\\ {ƒ}_{\sigma u}(lnZ)=-1874.31+210.91lnZ-8.46{\left(lnZ\right)}^{2}+0.13{\left(lnZ\right)}^{3}-1.04E-5{\left(lnZ\right)}^{4} \end{array}\right.$$

The correlation coefficient *R*, *R*-squared *R*^2^ and mean square error *MSE* are often used to measure the linear relationship between two variables. The larger the *R* and *R*^2^ value, the smaller the *MSE*, and the better the fitting effect. They are calculated as follows:

(19) $$R=\frac{\sum_{i=1}^{N}\left(X^{i}-\bar{X})\;(Y^{i}-\bar{Y}\right)}{\sqrt{\sum_{i=1}^{N}{\left(X^{i}-\bar{X}\right)}^{2}}\sqrt{\sum_{i=1}^{N}{\left(Y^{i}-\bar{Y}\right)}^{2}}}$$

(20) $$R^{2}=\frac{\sum_{i=1}^{N}{\left(X^{i}-\bar{Y}\right)}^{2}}{\sum_{i=1}^{N}{\left(Y^{i}-\bar{Y}\right)}^{2}}$$

(21) $$MSE=\frac{\sum_{i=1}^{N}{\left(Y^{i}-X^{i}\right)}^{2}}{N}$$

In the formula, $X^{i}$ is the value of the *i*th simulation; $Y^{i}$ is the *i*th test value; $\bar{X}$ is the average value of the simulation; $\bar{Y}$ is the average value of the test value. Substitute the experimental and simulation values of damage strain threshold *ε_th_*, damage resistance factor *S*, critical damage value *D*_1*c*_, and ultimate stress value *σ_u_* into Equations (19)–(21) for calculation, and obtain the data in Table 6.

It can be seen from Table 6 that the correlation coefficient *R* values obtained after fitting are all above 0.90. This shows that the established Cr5 alloy steel Lemaitre high-temperature damage model has high reliability. It can be seen from Figure 7 that the high-temperature Lemaitre damage model of the Cr5 alloy steel has a strong correlation with the actual test values [41]. This shows that the high-temperature Lemaitre damage model of Cr5 alloy steel can describe the damage characteristics of Cr5 alloy steel at different temperatures and strain rates in industrial production. By coupling and correcting the Lemaitre damage model with the Zener–Hollomon coefficient and the elastic modulus value *E* of the Cr5 alloy steel at different temperatures, it can provide an effective means of controlling the crack defects of Cr5 alloy steel roll products in the actual industry.

## 4. Verification Test of High-Temperature Damage Model

### 4.1. Finite Element Modelling of Tensile Process of Cr5 Alloy Steel

The reliability of the high-temperature damage model of the Cr5 alloy steel Lemaitre can effectively be verified by the finite element simulation technology. Using FORTRAN language, the Cr5 alloy high-temperature damage model was programmed, and it was embedded into Forge^®^ software through dynamic link library, and performed a numerical simulation of tensile test process of Cr5 alloy steel [42]. By comparing the simulation results with the test results, the accuracy of the high-temperature damage model of the Cr5 alloy steel Lemaitre can be verified.

**Figure 8:**
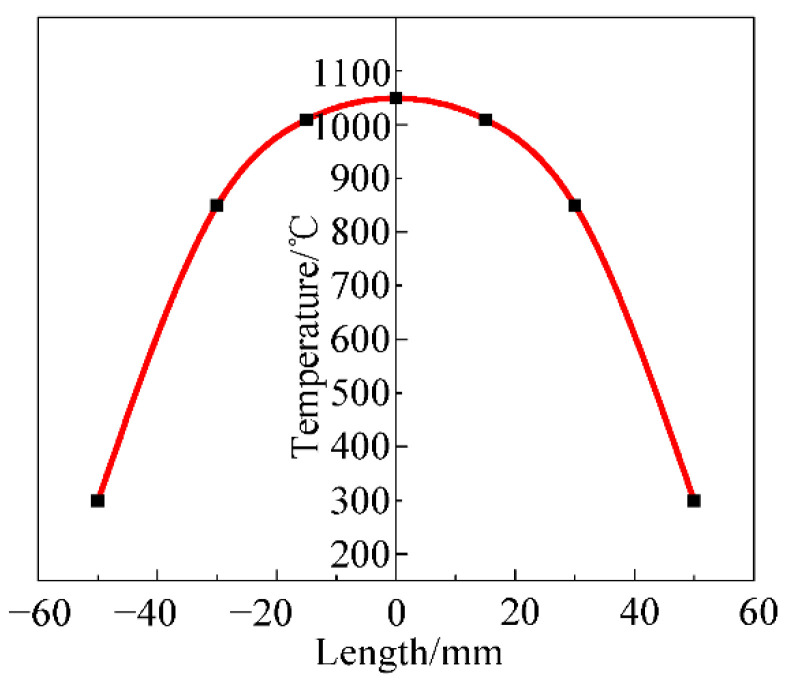
Cr5 alloy steel tensile specimen temperature distribution.

**Figure 9:**
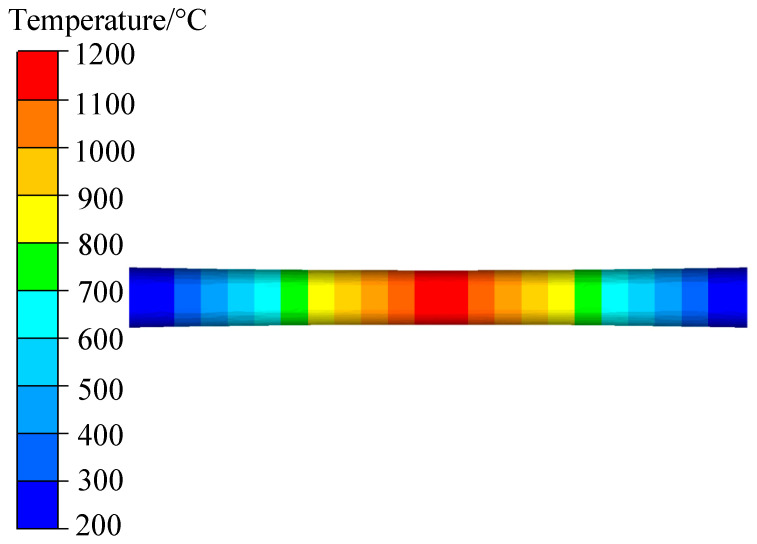
Cr5 alloy steel tensile simulation test modelling schematic diagram.

Since only the temperature of the centre of the sample can reach the specified temperature during the actual tensile test, the temperature on both sides gradually decreases and the magnitude of the temperature decrease increases with the distance from the centre of the sample. During the tensile test, a thermocouple wire was used to measure the temperature of the sample and the sample was kept at this temperature. The temperature distribution of the tensile sample with the length of the sample is shown in Figure 8. The length scale takes the centre of the sample as the origin. The temperature of the simulated tensile specimen was set according to the actual temperature distribution. The finite element geometric model and temperature distribution are shown in Figure 9. The mesh size is set to 0.5, the temperature and strain rate refer to the experimental conditions, the Coulomb friction coefficient is 0.3, the heat exchange coefficient is 2000 W/m^2^·K, and the emissivity is 0.88.

### 4.2. Verification of Lemaitre High-Temperature Damage Model of Cr5 Alloy Steel

**Figure 10:**
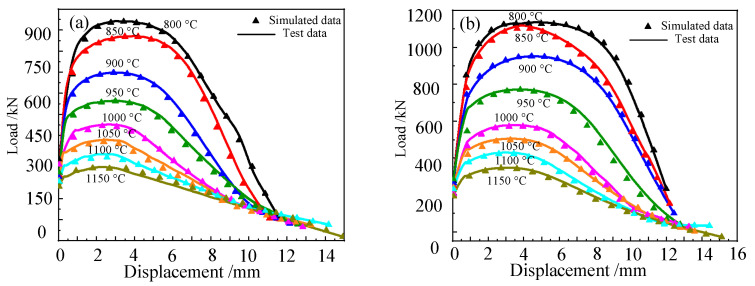

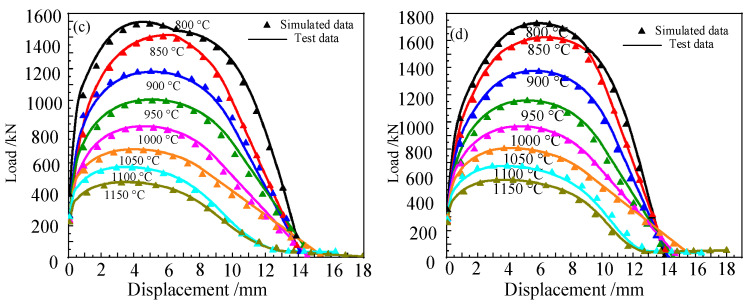
Comparison of displacement–load curves between the simulated tensile test and actual tensile test of Cr5 alloy steel: (**a**) $\dot{\varepsilon }$ = 0.01 s^−1^; (**b**) $\dot{\varepsilon }$ = 0.1 s^−1^; (**c**) $\dot{\varepsilon }$ = 1 s^−1^; (**d**) $\dot{\varepsilon }$ = 5 s^−1^.

By way of the observation and analysis of the digital simulation results of the tensile test, the displacement–load curves of the Cr5 alloy steel at various temperatures and strain rates are obtained, and the results obtained by digital simulation are compared with the actual tensile test results of the Cr5 alloy steel, as shown in Figure 10.

As can be seen from Figure 10, the displacement–load curve obtained by the tensile simulation test of Cr5 alloy steel is compared with the actual tensile test, and at 800 °C, 850 °C, 900 °C and 950 °C, they basically overlap; at 1000 °C, 1050 °C, 1100 °C and 1150 °C, the deviation is small. By comparison, it can be found that the displacement–load curve obtained by the tensile simulation test is basically consistent with the displacement–load curve obtained by the actual tensile test. The correlation coefficient *R*^2^ was used to compare the deviation between the experimental and simulated displacement–load curves. The simulation test data and the displacement–load data of the actual tensile test are brought into Formula (20). It was calculated that *R*^2^ = 0.9874.

**Figure 11:**
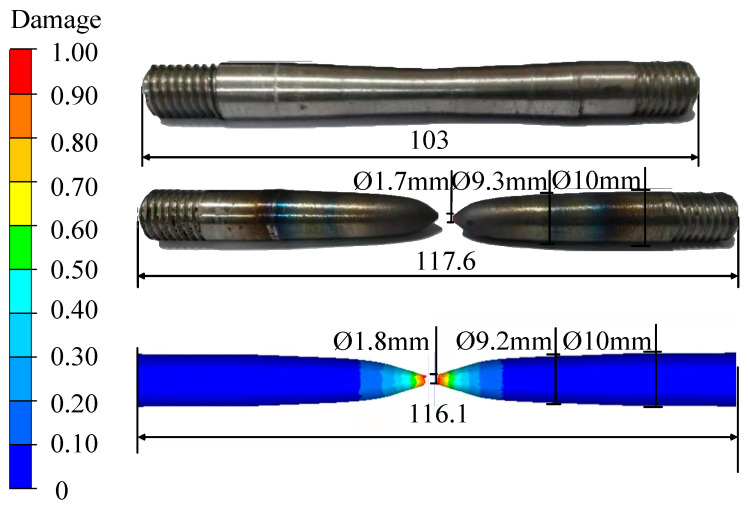
Schematic diagram of the comparison of gauge distance and tensile fracture between simulated tensile specimen and actual tensile specimen at 950 °C and 5 s^−1^ (unit: mm).

Figure 11 is a schematic diagram of the comparison of the gauge length and fracture of the simulated tensile specimen of Cr5 alloy steel at 950 °C with a strain rate of 5 s^−1^ and the actual tensile specimen.

As can be seen from Figure 11, based on the high-temperature damage model of Cr5 alloy steel Lemaitre, under the conditions of 950 °C and strain rate of 5 s^−1^, the fracture gauge length difference between the numerical simulation tensile specimen and the actual tensile specimen is 1.28%, the difference of the area reduction in the fracture surface is 1.1% and the difference of the area reduction at the centre between the fracture surface and the nearest large end face of the sample is 2.1%. The accuracy and reliability of the high-temperature damage model of Cr5 alloy steel Lemaitre are verified, and it shows that the high-temperature damage model is suitable for predicting the plastic damage of Cr5 alloy steel at different temperatures and strain rates. It can effectively prevent the occurrence of crack defects in the production of Cr5 rolls.

### 4.3. Damage Map of Lemaitre High-Temperature Damage Model

**Figure 12:**
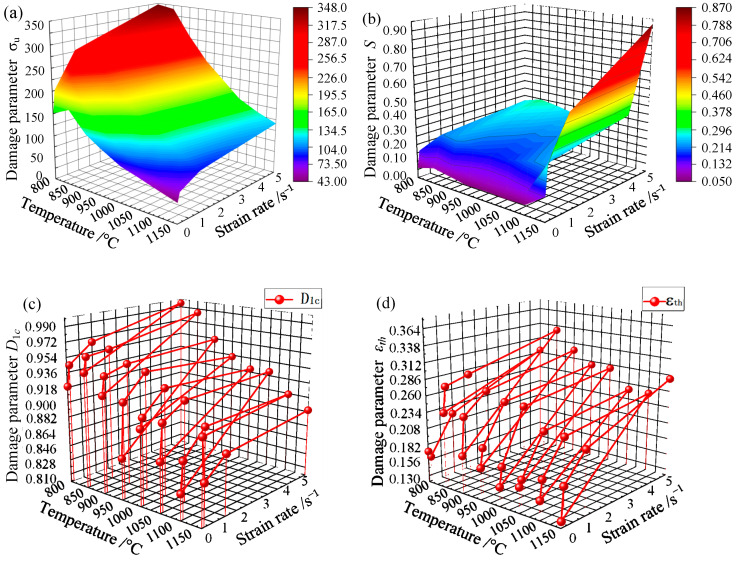
Damage diagram of Lemaitre damage parameters of Cr5 alloy steel: (**a**) surface diagram based on ultimate stress *σ_u_* with temperature and strain rate; (**b**) surface diagram based on anti-damage factor *S* with temperature and strain rate; (**c**) surface diagram based on critical damage value *D*_1*c*_ with temperature and strain rate; and (**d**) surface diagram based on damage strain threshold *ε_th_* with temperature and strain rate.

In order to have a more comprehensive understanding of the damage characteristics of Cr5 alloy steel and to more intuitively discern the relationship between the various damage parameter values of the Lemaitre damage model, the calculated damage parameter values of the Lemaitre high-temperature damage model at different temperatures and different strain rates of Cr5 alloy steel are plotted with their corresponding temperatures and strain rates. The damage map of the Lemaitre high-temperature damage model of the Cr5 alloy steel can be obtained, as shown in Figure 12.

As can be seen from Figure 12a, Cr5 alloy steel ultimate stress value *σ_u_* is inversely proportional to temperature and proportional to the strain rate. When the temperature is 800 °C and the strain rate is 0.01 s^−1^, the ultimate stress value *σ_u_* is 150.95 MPa; when the strain rate is increased to 5 s^−1^, *σ_u_* is 347.46 MPa; when the strain rate is 0.01 s^−1^ and the temperature is increased to 1150 °C, the ultimate stress value *σ_u_* is 43.28 MPa. It can be seen from the whole relationship diagram that the maximum value of the ultimate stress value *σ_u_* appears at 800 °C and 5 s^−1^, and the ultimate stress value *σ_u_* is 347.46 Mpa; the minimum value of the ultimate stress value *σ_u_* appears at 1150 °C, 0.01 s^−1^, and the ultimate stress value *σ_u_* at this time is 43.28 MPa.

It can be seen from Figure 12b that the damage resistance factor *S* of the Cr5 alloy steel is proportional to the temperature and inversely proportional to the strain rate. For example, when the strain rate is 0.01 s^−1^ and the temperature is 800 °C, the damage resistance factor *S* is 0.059 MPa, and the damage resistance factor *S* is 0.265 MPa when the strain rate is increased to 5 s^−1^; when the temperature is increased to 1150 °C, the damage resistance factor *S* is 0.134 MPa. It can be seen from the whole relationship diagram that the maximum value of the anti-damage factor *S* appears at 1150 °C, 5 s^−1^, and the anti-damage factor *S* at this time is 0.870 Mpa; the minimum value of the anti-damage factor *S* appears at 800 °C/0.01 s^−1^, and the damage resistance factor *S* is 0.059MPa.

It can be seen from Figure 12c that the critical damage value *D*_1*c*_ of the Cr5 alloy steel is basically inversely proportional to the temperature and proportional to the strain rate. For example, when the strain rate is 0.01 s^−1^ and the temperature is 800 °C, the critical damage *D*_1*c*_ is 0.919, and the critical damage *D*_1*c*_ is 0.995 when the strain rate is increased to 5 s^−1^; when the temperature is increased to 1150 °C, the critical damage *D*_1*c*_ at 1150 °C is 0.701. From the whole relationship diagram, it can be seen that the maximum value of critical damage *D*_1*c*_ occurs at 800°C/5 s^−1^ and the critical damage *D*_1*c*_ at this time is 0.995; the minimum value of critical damage *D*_1*c*_ occurs at 1150 °C/0.01 s^−1^, and the critical damage *D*_1*c*_ at 0.701.

It can be seen from Figure 12d that the damage strain threshold *ε_th_* of the Cr5 alloy steel is proportional to the strain rate and has little change under the influence of temperature. For example, when the strain rate is 0.01 s^−1^ and the temperature is 800 °C, the damage strain threshold *ε_th_* is 0.17, and the damage strain threshold *ε_th_* is 0.29 when the strain rate is increased to 5 s^−1^; when the strain rate is 0.01 s^−1^ and the temperature is 1150 °C, the damage strain threshold *ε_th_* is 0.13, and the damage strain threshold *ε_th_* is 0.28 when the strain rate is increased to 5 s^−1^. When the strain rate is 1 s^−1^, the damage strain threshold values *ε_th_* at 800–1150 °C are 0.22, 0.29, 0.27, 0.26, 0.26, 0.23, 0.23 and 0.22, and the change is not too obvious. It can be seen from the whole relationship diagram that the maximum damage strain threshold *ε_th_* appears at 850 °C/5 s^−1^, the damage strain threshold *ε_th_* at this time is 0.33; and the minimum damage strain threshold *ε_th_* appears at 1150 °C/0.01 s^−1^ and the damage strain threshold *ε_th_* at this time is 0.13.

From the damage diagram of the Lemaitre high-temperature damage model of the Cr5 alloy steel, it can be seen that when the temperature is constant, as the strain rate gradually increases, the critical damage value *D*_1*c*_ is basically positively correlated with the damage strain threshold value *ε_th_*. However, when the strain rate is constant, the correlation between the two is lower with the gradual increase in temperature; when the strain rate is constant, the ultimate stress *σ_u_* and the damage resistance factor *S* are basically negatively correlated with the gradual increase in temperature; when the temperature is constant, there is a positive correlation between the ultimate stress *σ_u_* and the damage resistance factor *S* as the strain rate gradually increases.

## 5. Conclusions

In order to accurately predict the crack defects of the Cr5 alloy steel during high-temperature forming. In the present study, the thermal tensile test and elastic modulus measurement experiment of Cr5 alloy under the conditions of 800–1150 °C and 0.01–5 s^−1^ were carried out, and the high-temperature damage model and damage diagram of the Cr5 alloy steel Lemaitre were established.

The elastic modulus *E* value of the Cr5 alloy steel decreases with the increase in temperature in the temperature range of 800–1150 °C. This is because when the temperature rises, the atomic spacing inside the material increases, the thermal motion of molecules increases, and then the ability to resist external elastic deformation is weakened. The test results show that the decrease in elastic modulus at high temperature increases the damage resistance factor *S*. In order to more accurately describe the effect of temperature on the elastic modulus, the functional relationship between the elastic modulus of the Cr5 alloy steel and temperature is determined.

Considering the influence of the temperature and strain rate on the damage of Cr5 alloy steel, Zener–Hollomon coefficient was introduced, the parameters of Lemaitre high-temperature damage model were measured, and the Lemaitre damage model of the Cr5 alloy steel at a high-temperature stage was founded.

The established high-temperature damage model was embedded in a Forge^®^ finite element software through the program’s secondary development method to carry out the numerical simulation calculation of the strength of the experimental samples. Comparing the difference between the simulation and the test displacement–load curve, the obtained correlation coefficient (*R*^2^) was 0.987. The fracture gauge length of the tensile specimen differed by 1.28% and the fracture morphology was basically consistent. This shows that the high-temperature damage model of the Cr5 alloy steel Lemaitre established in this paper has high accuracy in predicting the damage behaviour of this material. Finally, the damage map of Cr5 alloy steel at the high-temperature stage was constructed. The relationship between the damage parameters of the high-temperature damage model of Cr5 alloy steel and the deformation temperature and strain rate were analysed. This provides an effective method for controlling crack defects in the Cr5 alloy steel during high-temperature plastic deformation. In this paper, a high temperature damage model is established for Cr5 alloy steel. In the future, the production process of the Cr5 large roller will be simulated and verified in real industry.

## Figures and Tables

**Table 1:** Chemical compositions of Cr5 steel in wt%.

C	Mn	Si	Cr	Mo	V	Ni	Fe
0.52	0.453	0.547	4.56	0.478	0.129	0.426	93.1

**Table 2:** Fracture displacement of Cr5 alloy steel under different deformation conditions.

Fracture Displacement	800 °C	850 °C	900 °C	950 °C	1000 °C	1050 °C	1100 °C	1150 °C
0.01 s^−1^	11.52 mm	11.76 mm	12.14 mm	12.56 mm	12.83 mm	13.25 mm	14.18 mm	14.93 mm
0.1 s^−1^	11.86 mm	12.08 mm	12.44 mm	12.79 mm	13.28 mm	13.59 mm	14.43 mm	15.12 mm
1 s^−1^	13.89 mm	13.92 mm	14.12 mm	14.35 mm	14.55 mm	15.36 mm	16.27 mm	17.93 mm
5 s^−1^	14.02 mm	14.07 mm	14.32 mm	14.60 mm	14.69 mm	15.49 mm	16.43 mm	18.05 mm

**Table 3:** Determination of elastic modulus *E* values of Cr5 steel at different temperatures.

Temperature/°C	800	850	900	950	1000	1050	1100	1150
*E*/GPa	55	50	42	35	25	18	12	3.5

**Table 4:** Tensile properties, Lemaitre damage parameters and Zener–Hollomon coefficient of Cr5 steel at different temperatures and different strain rates.

Tensile Performance Parameters				Lemaitre Damage Parameters				Zener–Hollomon Coefficient Value			
T/°C	$\dot{\varepsilon }$/s^−1^	*R_m_*/MPa	*ε_m_*	*R_B_*/MPa	*ε_B_*	*D*_1*c*_	*σ_u_*_/MPa_	S/MPa	*ε_th_*	Z	LnZ
800	0.01	150.95	0.17	12.21	0.43	0.919	150.95	0.059	0.17	1.11 × 10^18^	41.55
800	0.1	201.02	0.16	51.45	0.48	0.944	201.02	0.158	0.16	1.11 × 10^19^	43.85
800	1	277.73	0.22	9.4	0.51	0.966	277.73	0.210	0.22	1.11 × 10^20^	46.16
800	5	347.46	0.29	1.58	0.53	0.995	347.46	0.265	0.29	5.56 × 10^20^	47.77
850	0.01	167.72	0.24	9.99	0.54	0.940	167.72	0.090	0.24	7.36 × 10^16^	38.84
850	0.1	235.59	0.38	9.92	0.57	0.958	235.59	0.110	0.28	7.36 × 10^17^	41.14
850	1	286.44	0.29	11.14	0.56	0.961	286.44	0.230	0.29	7.36 × 10^18^	43.44
850	5	341.03	0.33	5.22	0.58	0.985	341.03	0.295	0.33	3.68 × 10^19^	45.05
900	0.01	126.17	0.18	10.39	0.53	0.918	126.17	0.072	0.18	6.58 × 10^15^	36.42
900	0.1	180.98	0.24	10.88	0.55	0.940	180.98	0.129	0.24	6.58 × 10^16^	38.73
900	1	230.9	0.27	12.07	0.56	0.948	230.9	0.194	0.27	6.58 × 10^17^	41.03
900	5	281.35	0.3	13.13	0.58	0.953	281.35	0.277	0.3	3.29 × 10^18^	42.64
950	0.01	101.07	0.17	15.18	0.48	0.850	101.07	0.053	0.17	7.59 × 10^14^	34.26
950	0.1	141.61	0.2	12.053	0.54	0.915	141.61	0.106	0.2	7.59 × 10^15^	36.57
950	1	193.69	0.26	11.03	0.57	0.943	193.69	0.176	0.26	7.59 × 10^16^	38.87
950	5	230.58	0.28	15.14	0.59	0.934	230.58	0.252	0.28	3.79 × 10^17^	40.48
1000	0.01	79.457	0.15	8.66	0.55	0.891	79.457	0.057	0.15	1.09 × 10^14^	32.32
1000	0.1	107.52	0.18	10.4	0.56	0.903	107.52	0.097	0.18	1.09 × 10^15^	34.62
1000	1	162.24	0.26	11.57	0.58	0.929	162.24	0.181	0.26	1.09 × 10^16^	36.93
1000	5	192.58	0.28	15.08	0.58	0.922	192.58	0.241	0.28	5.44 × 10^16^	38.54
1050	0.01	67.786	0.16	9.51	0.51	0.860	67.786	0.052	0.16	1.88 × 10^13^	30.56
1050	0.1	91.311	0.17	8.87	0.57	0.903	91.311	0.103	0.17	1.88 × 10^14^	32.86
1050	1	132.57	0.23	10.65	0.62	0.920	132.57	0.207	0.23	1.88 × 10^15^	35.17
1050	5	154.45	0.25	11.93	0.62	0.923	154.45	0.266	0.25	9.37 × 10^15^	36.78
1100	0.01	55.217	0.15	9.32	0.51	0.831	55.217	0.055	0.15	3.79 × 10^12^	28.97
1100	0.1	78.036	0.18	10.37	0.59	0.867	78.036	0.120	0.18	3.79 × 10^13^	31.27
1100	1	111.01	0.23	11.54	0.63	0.896	111.01	0.229	0.23	3.79 × 10^14^	33.57
1100	5	132.74	0.25	13.35	0.64	0.899	132.74	0.318	0.25	1.89 × 10^15^	35.19
1150	0.01	43.281	0.13	12.94	0.48	0.701	43.281	0.134	0.13	8.81 × 10^11^	27.5
1150	0.1	63.466	0.18	9.55	0.61	0.850	63.466	0.291	0.18	8.81 × 10^12^	29.81
1150	1	87.469	0.22	11.18	0.64	0.872	87.469	0.526	0.22	8.81 × 10^13^	32.11
1150	5	116.04	0.28	13.42	0.68	0.884	116.04	0.870	0.28	4.40 × 10^14^	33.72

**Table 5:** Values of the coefficients of the modified Lemaitre damage model for Cr5 steel.

The Values of the Coefficients of the Lemaitre Damage Model	A	B	C	D	E
Damage strain threshold *ε_th_*	1.27	−0.14	0.006	−7.99 × 10^−5^	5.81 × 10^−9^
Anti-damage factor *S*	5.48	−0.61	−0.025	−4.43 × 10^−4^	2.94 × 10^−6^
Critical damage value under uniaxial tension *D*_1*c*_	1.59	0.13	0.66	0	0
Ultimate stress value under uniaxial tension *σ_u_*	−1874.31	210.91	−8.46	0.13	−1.04 × 10^−5^

**Table 6:** The coefficients of the modified Lemaitre damage model for Cr5 steel.

Difference Analysis of Coefficient Values	Correlation Coefficient *R*	*R*-Squared R^2^	Mean Square Error *MSE*
**Damage strain threshold *ε_th_***	0.92	0.91	0.37
**Anti-injury factor *S***	0.93	0.92	22.01
**Critical damage value *D*_1*c*_**	0.92	0.91	8.99
**Ultimate stress value *σ_u_***	0.98	0.98	46.12

## Data Availability

Not applicable.
